# False Positive Babesia microti Result in New-Onset Systemic Lupus Erythematosus Manifesting With Febrile Illness

**DOI:** 10.7759/cureus.73740

**Published:** 2024-11-15

**Authors:** Anna Laskova, Bohdan Syritsa, Bing Han, Jian Jeff Fu

**Affiliations:** 1 Hospital Medicine, Reading Hospital, Tower Health, West Reading, USA; 2 Pathology, Reading Hospital, Tower Health, West Reading, USA

**Keywords:** acute babesiosis, babesia microti antibody test, false positive serology, febrile illness, systemic lupus erythematosus

## Abstract

False positive serologic results are common in systemic lupus erythematosus (SLE) due to the presence of autoantibodies. We present a case of a young patient initially suspected of having a tick-borne disease with a false positive Babesia microti antibody result, and later diagnosed with SLE. Acute babesiosis was excluded after additional laboratory tests such as Babesia polymerase chain reaction (PCR) and blood smear for parasites. The patient’s symptoms were then thought to be a new manifestation of SLE and prompted the initiation of systemic steroids with subsequent improvement. False positive serologic Babesia microti test result was attributed to SLE autoantibodies.

## Introduction

Systemic lupus erythematosus (SLE) is an autoimmune disease that can lead to various symptoms and present with mucocutaneous, hematological, gastrointestinal, renal, cardiovascular, and other organ system involvement. There are no definite diagnostic criteria. Several classification criteria were initially developed for research purposes, and are now used to help define which patients are more likely to have SLE. SLE is often associated with circulating antinuclear antibodies; for example, anti-double stranded DNA antibodies have been described as specific for SLE [1].

False positive serologic test results are common in patients with autoimmune diseases including SLE. Historically, false positive syphilis test results in patients with SLE have been studied extensively since the 1950s. Both nontreponemal and treponemal tests can be false positive in patients with SLE. Nontreponemal tests such as rapid plasma reagin use cardiolipin as the main antigen; anticardiolipin antibodies are common in SLE [2]. A beaded pattern of fluorescent treponemal antibody-absorption (FTA-ABS) test was strongly associated with connective tissue disorders [3]. Similarly, both enzyme-linked immunosorbent assay (ELISA) and Western blot HIV results can be false positive in lupus [4]. It was thought to be caused by cross-reactivity between lupus autoantibodies and HIV antigens. In addition, false positive Lyme disease results, cytomegalovirus (CMV), mumps, and, more recently, COVID-19 were studied [5]. Our case highlights a false positive Babesia test result in new-onset SLE.

## Case presentation

A 24-year-old male with a remote history of biopsy-proven eosinophilic esophagitis developed dysuria and presented to urgent care. The patient was diagnosed with acute cystitis and prescribed a course of trimethoprim-sulfamethoxazole. Upon completion of the seven days of the medication, the patient developed fever, generalized myalgia, arthralgia, and diffuse alopecia, and presented to the emergency department. Vitals signs were as follows: blood pressure 114/73 mm Hg, heart rate 69/min, respiratory rate 22/min, temperature 103.8 F, oxygen saturation (SpO2) 96% on room air. Physical examination was unremarkable. Laboratory results revealed pancytopenia, elevated transaminases, and elevated lactate dehydrogenase (Table 1). Haptoglobin, total, and direct bilirubin levels were within normal limits. There was no evidence of intravascular hemolysis on blood smear.

**Table 1:** Pertinent laboratory test results on admission. AST: aspartate aminotransferase ALT: alanine aminotransferase WBC: white blood cells ANC: absolute neutrophil count

Test	Result	Reference range
AST (peak)	359	<34 U/L
ALT (peak)	367	10-49 U/L
WBC	1*10^9^	4.8-10.8*10^9^/L
ANC	570	2000-8000 neutrophils/mcL
Platelets	103*10^9^	130-400*10^9^/L
Hemoglobin	10.7	14-17.5 g/L
Lactate dehydrogenase	343	125-220 IU/L

**Figure 1:**
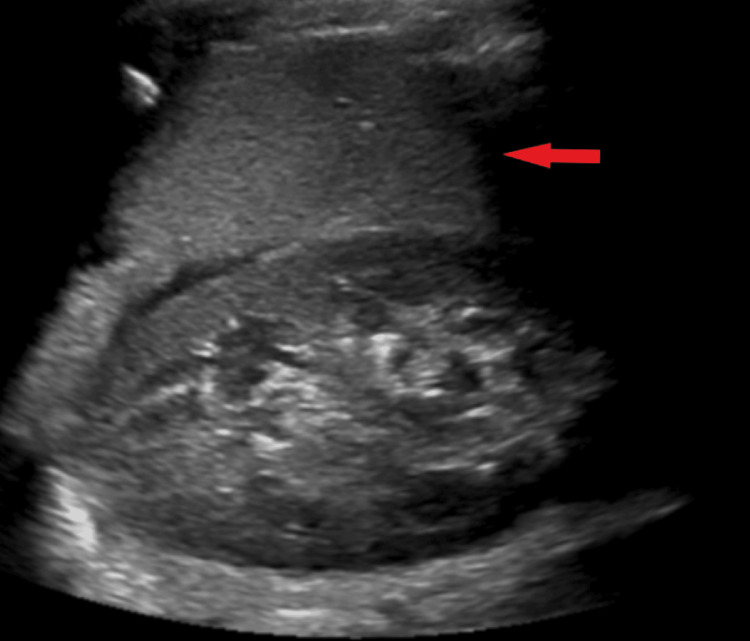
The spleen was measured at 14.2 cm in craniocaudal dimension on abdominal ultrasound corresponding with mild splenomegaly (red arrow).

Due to fever and leukopenia, sepsis was suspected. Multiple broad-spectrum antimicrobial agents were used throughout the hospitalization, including piperacillin-tazobactam, vancomycin, doxycycline, and azithromycin. Blood and urine cultures were negative. Abdominal ultrasound revealed splenomegaly (Figure 1).

The acute viral hepatitis panel was negative. The tick-borne disease panel was remarkable for positive Babesia microti serology. The Babesia microti IgG titer was elevated at 1:64 and rose to 1:512 three days later. However, the Babesia microti IgM titer remained normal on both days (<1:20). A blood smear for parasites was negative twice. The Babesia polymerase chain reaction (PCR) test was negative. Acute babesiosis was ruled out. In view of elevated transaminases of unclear etiology, pancytopenia, and febrile illness, antinuclear antibody panel, C3, and C4 complement levels were checked. The results were remarkable for elevated antinuclear antibody titer, anti-double stranded and anti-histone antibodies, and decreased complement levels (Table 2).

**Table 2:** Pertinent rheumatological test results.

Test	Result	Reference range
Antinuclear antibodies pattern 1 titer	1:80	<1:40
Antinuclear antibodies pattern 2 titer	1:1280	<1:40
Anti-double stranded DNA antibodies	1642	< or = 4 IU/ml
Anti-histone antibody	6.8	<1 U
C3 complement	31	82-185 mg/dL
C4 complement	10.3	15-53 mg/dL

**Figure 2:**
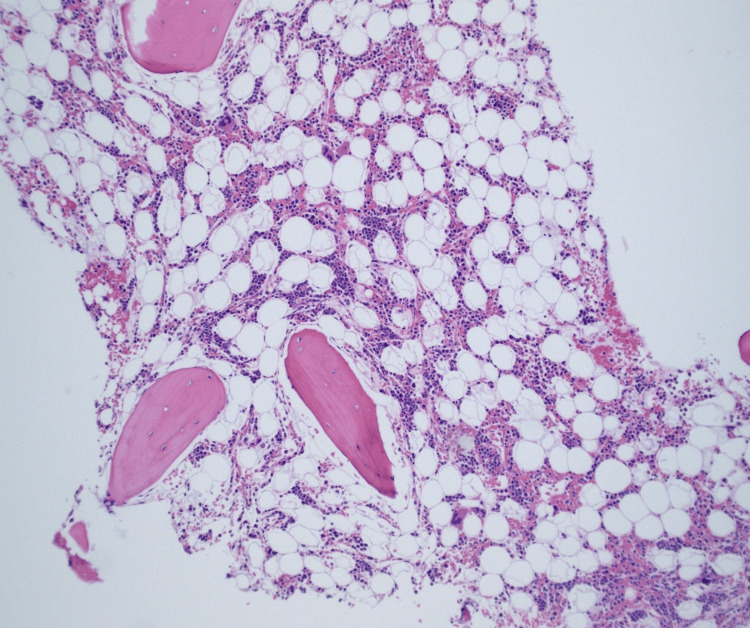
Bone marrow biopsy revealed significant hypocellularity.

The patient underwent a bone marrow biopsy that revealed marked hypocellularity (Figure 2). Flow cytometry showed no evidence of neoplasm. The Hematology team raised the possibility of hemophagocytic lymphohistiocytosis (HLH). Interleukin-2 receptor alpha chain/CD 25 was significantly elevated at 4206 (normal range 532-1891 pg/ml). However, the bone marrow biopsy result did not reveal hemophagocytosis. The patient met some but not all criteria for HLH.

**Figure 3:**
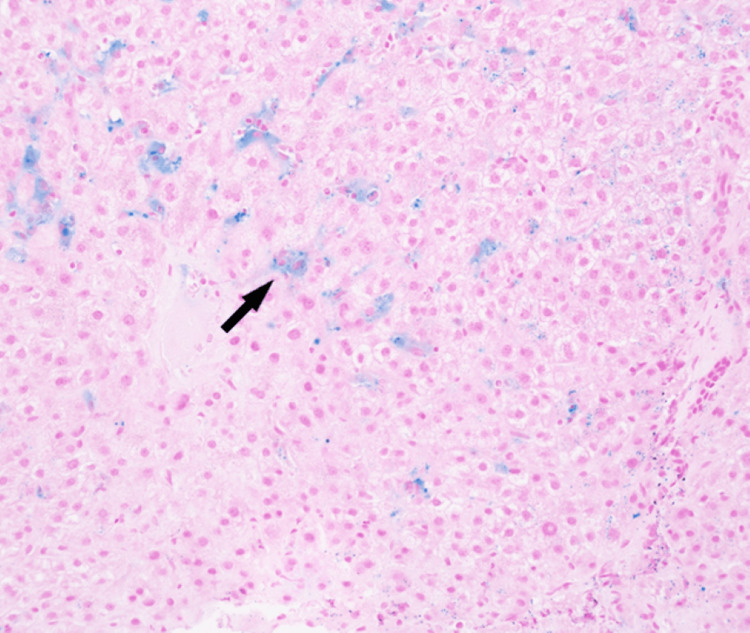
The liver biopsy iron stain demonstrated periportal intracellular iron deposition seen as blue granules (black arrow). The liver architecture was normal.

Persistent elevation of transaminases warranted further investigation. The ceruloplasmin and alpha-1-antitrypsin levels were normal. A liver biopsy showed findings suggestive of early-stage hemochromatosis (HFE) (Figure 3). However, the genetic testing for HFE was negative.

Antimicrobial agents were discontinued. Rheumatology was consulted, as the patient was suspected of having SLE. Methylprednisolone 125 mg IV daily was initiated on the 14th day of the hospitalization. Pancytopenia improved, and transaminase levels decreased significantly (Table 3). Methylprednisolone was weaned down and transitioned to oral prednisone 20 mg daily on the 19th day of the hospitalization. The patient’s symptoms resolved, and the patient was discharged home with subsequent follow-up with Rheumatology. The patient was recommended to avoid trimethoprim-sulfamethoxazole use indefinitely since it was also questioned whether or not the patient had medication-induced lupus.

**Table 3:** Laboratory test results on the ninth day after steroid treatment initiation. AST: aspartate aminotransferase ALT: alanine aminotransferase WBC: white blood cells

Test	Result	Reference range
AST	55	<34 U/L
ALT	154	10-49 U/L
WBC	4.8*10^^9^	4.8-10.8*10^^9^/L
Platelets	184*10^^9^	130-400*10^^9^/L
Hemoglobin	10.6	14-17.5 g/L

## Discussion

This young patient presented with significant nonspecific symptoms and at the beginning received treatment targeting possible infection. Initially, a tick-borne disease was suspected. Interestingly, a Babesia microti antibody titer was checked twice; the Babesia microti IgG titer rose from 1:64 to 1:512 in three days, while the Babesia microti IgM titer remained normal on both days. We expect an elevated Babesia microti IgM titer in acute or recent infection. Also, if the elevated Babesia microti IgG titer were from a past or recent infection, we would not have seen a rising titer from 1:64 to 1:512 in three days. A false positive result was suspected due to a discrepancy between IgG and IgM results and a negative blood smear result. Moreover, the Babesia PCR send-out test demonstrated a negative result, which ruled out acute babesiosis. It was a crucial diagnostic step because the clinical presentation had some features of acute babesiosis such as fever, splenomegaly, and anemia. Such abnormal Babesia IgG result was attributed to the known impact of lupus antibodies on serologic testing.

Diagnosis of acute babesiosis usually involves the demonstration of parasites on blood smears. The blood parasite smear cannot always detect parasites in case of low levels of parasitemia. In this case, the Infectious Diseases Society of America (IDSA) recommends PCR for diagnosis of acute babesiosis rather than antibody testing. An indirect fluorescent antibody (IFA) test is still routinely used to detect antibodies in blood as a part of the tick-borne disease panel. A Babesia microti IgG titer of 1:1024 or more or a four-fold rise in Babesia microti IgG titer can suggest active or recent Babesia microti infection. However, a positive Babesia microti antibody test should be confirmed by detecting parasites on the blood smear or PCR according to IDSA [6]. Acute babesiosis was ruled out in our patient by both negative parasite smear and Babesia PCR test.

We would like to point out other features of the clinical presentation, although it is not the main focus of our case report. The liver biopsy revealed findings suggestive of early-stage hemochromatosis due to periportal intracellular iron deposition, though the patient lacked the HFE gene. The patient did not have evidence of intravascular or extravascular hemolysis, so it was unlikely to cause secondary iron overload. Such secondary hemochromatosis could be due to systemic inflammation in the setting of SLE as previously highlighted in the literature [7]. In our patient, the inflammatory condition called HLH was suspected though not proven. HLH is a syndrome characterized by significant systemic inflammation caused by either genetic mutation (primary HLH) or acquired in the setting of autoimmune disease, malignancy, or infection (secondary HLH) [8]. Specifically, the patient met some features of HLH such as pancytopenia, fever, splenomegaly, elevated ferritin, and elevated interleukin-2 receptor alpha chain/CD 25. However, the patient did not meet all the diagnostic criteria of HLH, because we could not demonstrate the presence of hemophagocytosis in the bone marrow or liver due to technical limitations of the specimen.

The strongly positive anti-histone antibody result raised the possibility of the differential diagnosis of medication-induced lupus. Therefore, the patient was recommended to avoid trimethoprim-sulfamethoxazole in the future. Although the patient had elevated anti-histone antibodies, these antibodies can also be present in SLE. Meanwhile, anti-double-stranded DNA antibodies are rarely elevated in medication-induced lupus. Finally, even though the patient developed symptoms after taking trimethoprim-sulfamethoxazole, this happened shortly after the medication was started but the symptoms of medication-induced lupus usually occur at least four weeks after taking the culprit medication [9]. For these reasons, the most likely diagnosis at hospital discharge was SLE.

## Conclusions

The infectious workup is crucial in febrile illness with systemic features. Infection should be ruled out before the use of immunosuppressive agents such as steroids in suspected cases of an autoimmune disease. It is important to remember that autoimmune conditions such as SLE can lead to false positive serologic results that can be misleading. The Babesia antibody test should not be used to routinely diagnose acute babesiosis. Instead, demonstrating parasites on blood smear or positive PCR result should confirm acute babesiosis. 
